# The Role of Cerebral Hypoperfusion in Multiple Sclerosis (ROCHIMS) Trial in Multiple Sclerosis: Insights From Negative Results

**DOI:** 10.3389/fneur.2020.00674

**Published:** 2020-07-14

**Authors:** Stéphanie Hostenbach, Hubert Raeymaekers, Peter Van Schuerbeek, Anne-Marie Vanbinst, Wilfried Cools, Jacques De Keyser, Miguel D'Haeseleer

**Affiliations:** ^1^Department of Neurology, Universitair Ziekenhuis Brussel, Brussels, Belgium; ^2^Center for Neurosciences, Vrije Universiteit Brussel, Brussels, Belgium; ^3^Department of Radiology and Medical Physics, Universitair Ziekenhuis Brussel, Brussels, Belgium; ^4^Interfaculty Center Data Processing and Statistics, Vrije Universiteit Brussel, Brussels, Belgium; ^5^Department of Neurology, Universitair Medisch Centrum Groningen, Groningen, Netherlands; ^6^National Multiple Sclerosis Centrum, Melsbroek, Belgium

**Keywords:** multiple sclerosis, cerebral blood flow, endothelin-1, N-acetylaspartate, bosentan, normal appearing white matter

## Abstract

**Background:** Accumulating evidence indicates that mitochondrial energy failure is involved in the progressive axonal degeneration in multiple sclerosis (MS). In patients with MS, it has been shown that both levels of N-acetylaspartate (NAA), which is a marker of axonal mitochondrial energy, and cerebral blood flow (CBF) are reduced in cerebral normal appearing white matter (NAWM). The latter is likely due to the vasoconstrictive action of endothelin-1 (ET-1) produced by reactive astrocytes, which is triggered by local proinflammatory cytokines. A preliminary study in patients with MS showed that CBF could be restored to normal values after a single dose of 62.5 mg of the ET-1 antagonist bosentan.

**Objective:** To investigate whether restoring CBF in patients with relapsing remitting MS (RRMS) increases levels of NAA in cerebral NAWM and improves clinical symptoms.

**Methods:** 27 RRMS patients were included in a 4 weeks proof-of-concept, randomized, double-blind placebo-controlled trial (ROCHIMS) to investigate whether bosentan 62.5 mg twice daily could increase the NAA/creatine (NAA/Cr) ratio in NAWM of the centrum semiovale. Magnetic resonance imaging (MRI) assessing CBF and NAA/Cr, and clinical evaluations were performed at baseline and at end of study. Separately from the clinical trial, 10 healthy controls underwent the same baseline multimodal brain MRI protocol as the MS patients.

**Results:** Eleven patients in the bosentan arm and thirteen patients in the placebo arm completed the study. Bosentan did not increase CBF. However, we found that CBF in the patients was not different from that of the healthy controls. There were no effects on NAA levels and clinical symptoms.

**Conclusions:** Our study showed that CBF in RRMS patients is not always decreased and that bosentan has no effect when CBF values are within the normal range. We hypothesize that in our patients there was no significant astrocytic production of ET-1 because they had a mild disease course, with minimal local inflammatory activity. Future studies with bosentan in MS should focus on patients with elevated ET-1 levels in cerebrospinal fluid or blood.

## Introduction

Multiple sclerosis (MS) is a chronic inflammatory and neurodegenerative disorder of the central nervous system (CNS). The disease pathology is characterized by the occurrence of focal inflammatory demyelinating lesions responsible for relapses, and a progressive axonal degeneration responsible for a more slowly decline of neurologic functions (1). Inflammatory immune responses play an important role in the pathogenesis of the focal lesions. However, less is known about the mechanisms underlying progressive axonal degeneration, which for most MS patients seems to be the primary determinant of long-term disability (2).

There is increasing evidence that mitochondrial energy failure and oxidative stress are involved in the progressive axonal degeneration of MS (3, 4). In cerebral white matter, the level of N-acetylaspartate (NAA), an amino acid synthesized by mitochondria in neurons, is considered a marker of both axonal mitochondrial activity and axonal integrity (5). ^1^H-magnetic resonance spectroscopy (^1^H- MRS) studies of cerebral normal-appearing white matter (NAWM) in patients with MS found reduced NAA levels, as compared to control subjects (1, 6). In addition, a number of studies found that cerebral blood flow (CBF) is impaired in NAWM of patients with relapsing-remitting MS (RRMS), primary progressive MS and also in clinically isolated syndromes suggestive for MS (CIS-MS) suggesting that this is independent of the disease course of MS and can already be present in early phases of the disease (3, 7–12). In animal models, chronically induced cerebral hypoperfusion leads to mitochondrial energy failure, oxidative stress, and axonal degeneration (13). There are several lines of evidence that ATP synthesis and NAA synthesis are coupled, and a number of experiments have shown a decrease in NAA levels when brain energy metabolism is disrupted (5). Thus, in patients with MS, there might be a link between reduced CBF and axonal mitochondrial energy failure (5).

An increased production of the potent vasoconstrictive agent endothelin-1 (ET-1) by reactive astrocytes in focal demyelinated lesions appears to play an important role in reducing CBF in patients with MS (3). ET_A_ receptor activation on vascular smooth muscle cells by ET-1 induces a dose-dependent vasoconstriction of human cerebral arteries, which can be antagonized by the ET-1 receptor antagonist bosentan (14). D'Haeseleer et al. found that the administration of a single 62.5 mg dose of bosentan in MS patients restored CBF to levels similar to healthy controls (3). As an extension of this study, we performed the ROCHIMS (Role of Cerebral Hypoperfusion in Multiple Sclerosis) trial, which was a non-commercial proof-of-concept, placebo-controlled double-blind, randomized trial investigating whether in RRMS patients a more prolonged treatment with 62.5 mg bosentan twice daily during ±28 days results in a sustained restoration of CBF, and whether this enhances NAA levels in NAWM, reflecting an improvement of axonal mitochondrial metabolism. Furthermore, we wanted to explore if the restoration of CBF has an influence on clinical symptoms.

However, the results of our study were negative, but no less interesting as it further contributes to a better understanding of the role of CBF in the pathophysiology of MS.

## Methods

### Subjects and Procedures

The ROCHIMS trial was conducted at the Universitair Ziekenhuis (UZ) Brussel, Belgium, between October 2017 and March 2019. The protocol of the study has been described in detail in TRIALS (15). The study was registered by the European Union Drug Regulating Authorities (Eudra-CT 2017-001253-13) and was approved at national level by the Federaal Agentschap Voor Geneesmiddelen en Gezondheidsproducten (FAGG, Belgium) as well as by the ethics committee of the UZ Brussel. All patients gave written informed consent.

Patients with a diagnosis of RRMS, according to the 2010 revised McDonald Criteria (16), aged above 18 years and with an Expanded Disability Status Score (EDSS) less or equal than 4 were enrolled in the study. The exclusion criteria were an MS relapse within the last 3 months before inclusion, pregnancy or lactation, major liver dysfunction, use of cyclosporine A or glibenclamide, or allergy to bosentan. For sexually active female patients with reproductive potential, use of reliable means of contraception was required.

Randomization of the patients was performed on day 0 with “research randomizer” (https://randomizer.org), using 5 sets of 6 numbers in order to achieve an equal distribution between the active product (bosentan) and placebo. Bosentan tablets of 62.5 mg were provided by Actelion Pharmaceuticals Belgium NV (Mechelen, Belgium) and identical-looking placebo tablets were produced by the Hospital Pharmacy of the UZ Brussel.

At baseline, all patients underwent multimodal brain MRI consisting of a high-resolution anatomical scan, 3D fluid-attenuated inversion recovery, arterial spin labeling (ASL) and ^1^H MRS on a 3 Tesla machine (GE, MR 750W Discovery). Total scanning time was about 30 min. The regions of interest for CBF and NAA/creatine (NAA/Cr) quantification were the left and right centrum semiovale NAWM. The final results represent the average of the measurements in left and right centrum semiovale. All further technical details, including the software used for analysis of the ASL data and spectroscopic data are described in TRIALS (15).

All patients were assessed for a number of clinical parameters (a) fatigue, scored by the Fatigue Severity Scale (FSS), Modified Fatigue Impact Scale-5-item version (MFIS-5) and Fatigue Scale for Motor and Cognitive functions (FSMC), (b) depression by the Beck Depression Inventory-II, (c) a Brief International Cognitive Assessment for Multiple Sclerosis (BICAMS) consisting of Symbol Digit Modality Test (SDMT), California Verbal Learning Test (CVLT-II), and Brief Visuospatial Memory Test-Revised (BVMT-R), and (d) motor function of lower limbs by the Timed 25 Foot Walk Test (T25FWT) and of the upper limbs by the 9-Hole Peg Test (9HPT). All these tests are described in detail in TRIALS (15).

The daily intake of two tablets of study medication (one tablet at 8:00 a.m. and one tablet at 8:00 p.m.) started on day 1 and continued until day 28 (±2 days).

All evaluations described above were repeated at end of study. On the evaluation days, participants were asked to refrain from alcohol or caffeine, as well as not to smoke. Two weeks after ending the trial, participants were called at home to see if any side effects had occurred during the trial.

Not part of the ROCHIMS trial, 10 healthy controls underwent the same baseline multimodal brain MRI protocol as the patients with MS. This study was also approved by the ethics committee of the UZ Brussel. All controls gave written informed consent.

### Outcomes

The primary outcome of the ROCHIMS study was the change in NAA/Cr ratio in centrum semiovale NAWM, reflecting changes in axonal mitochondrial metabolism. Secondary clinical endpoints were the differences in change in fatigue, cognition, depression, and motor function, between the two treatment groups.

### Statistical Analyses

Sample size calculation was based on the study of Steen et al. NAA/Cr in centrum semiovale NAWM was found to be 10% lower in MS patients compared to healthy controls (6). It was estimated that 12 patients in each group would be needed to detect a 10% increase in NAA/Cr in centrum semiovale NAWM in the bosentan group compared to the placebo group (alpha value = 0.05, power 90%). Taking into account asymptomatic efficiency loss and potential dropouts, inclusion of 15 patients in each group were deemed sufficient.

The Wilcoxon signed-rank test for paired samples compared differences of parameters within one treatment group, and the Mann–Whitney *U*-test to compared differences between the groups. The level for statistical significance was defined as *p* < 0.05.

## Results

**Figure 1:**
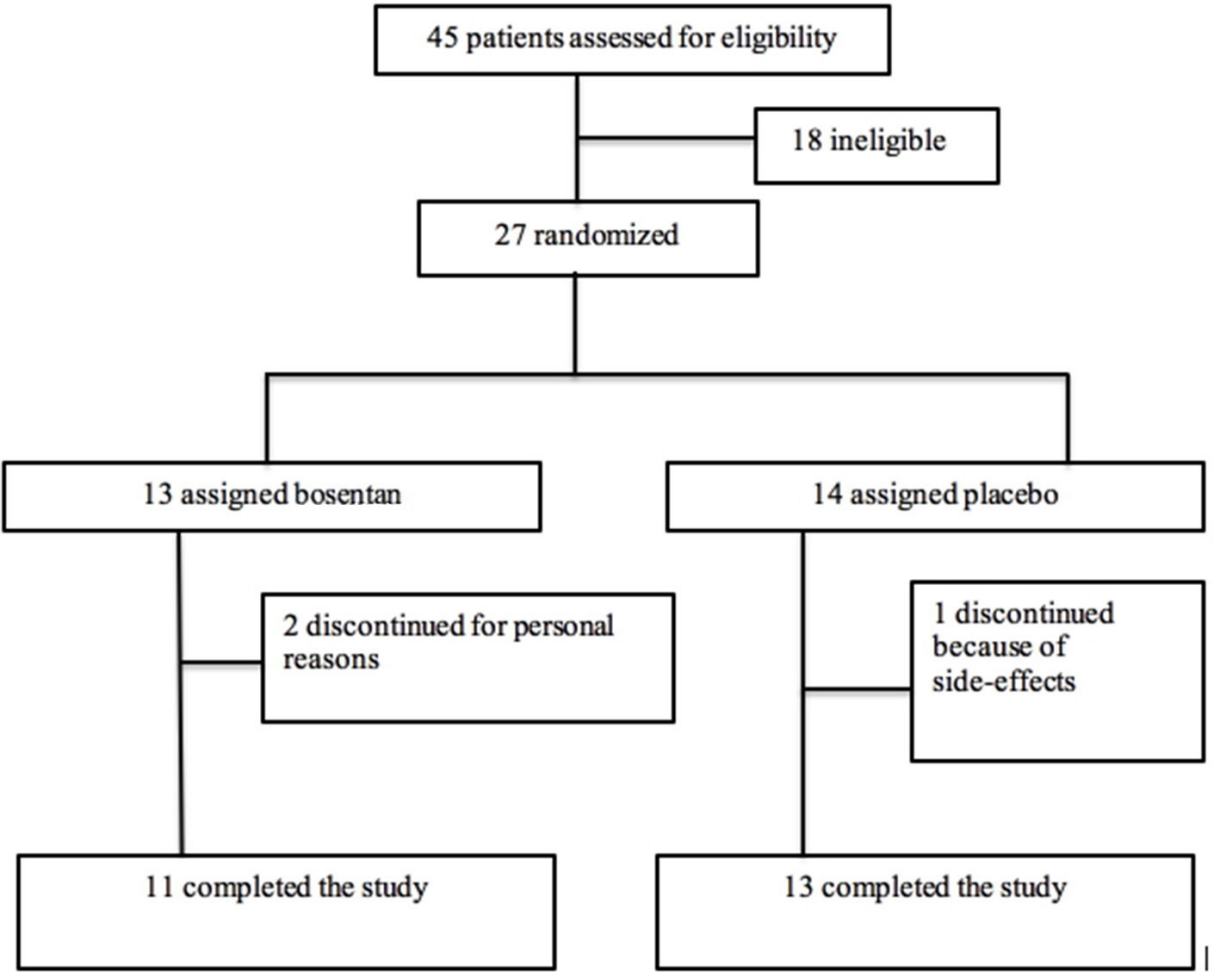
Consort Diagram of the ROCHIMS trial.

A total number of 27 patients with RRMS were randomly assigned to either bosentan (*n* = 13) or placebo (*n* = 14). The Consort Diagram is shown in Figure 1.

Two patients in the bosentan group and one patient in the placebo did not complete the study.

Demographic characteristics and baseline parameters of the RRMS patients are shown in Table 1. There were no significant differences between the two treatment groups, except for age.

**Table 1:** Demographics and baseline values of the RRMS patients who completed the study.

	**Bosentan (*n* = 11)**	**Placebo (*n* = 13)**
Sex (female)	6 (54 %)	10 (77%)
AGE^*^ (YEARS)		
Mean (*SD*)	44 (8)	38 (6)
Median (Q1, Q3)	45 (38, 53)	37 (34, 42)
**DISEASE DURATION (YEARS)**		
Mean (*SD*)	6.9 (5.1)	5.8 (4.7)
Median (Q1, Q3)	5 (4, 14)	3 (2, 9)
**EDSS**		
Mean (*SD*)	1.55 (1.17)	1.19 (1.15)
Median (Q1, Q3)	2 (0, 2)	1.5 (0, 2)
**DISEASE-MODIFYING TREATMENT**		
Yes	9 (81.82%)	12 (92.31%)
Natalizumab	2	4
Dimethyl fumarate	2	2
Interferon	1	3
Glatiramer acetate	0	0
Teriflunomide	2	1
Alemtuzumab	1	0
Fingolimod	1	2
**NAA/CR**		
Mean (*SD*)	2 (0.19)	2.04 (0.19)
Median (Q1, Q3)	1.98 (1.87, 2.11)	1.96 (1.89, 2.27)
**CBF (ml/100 g/min)**		
Mean (*SD*)	26.54 (3.81)	26.04 (2.70)
Median (Q1, Q3)	26.78 (23.21, 28.37)	26.27 (24.26, 28.32)
**FSS**		
Mean (*SD*)	39.18 (12.43)	34.62 (8.80)
Median (Q1, Q3)	45 (30, 58)	34 (27, 40.5)
**MFIS**		
Mean (*SD*)	9.73 (4.57)	10.15 (2.23)
Median (Q1,Q3)	11 (4, 16)	10 (8.5, 12)
**FSMC**		
Mean (*SD*)	54.40 (20.75)	56.23 (14.32)
Median (Q1, Q3)	60 (34.75, 67.50)	53 (44.5, 71)
**BDI-II**		
Mean (*SD*)	9.91 (7.05)	9.54 (6.65)
Median (Q1, Q3)	9 (4, 16)	9 (5, 13)
**SDMT**		
Mean (*SD*)	51.27 (11.42)	54.85 (7.16)
Median (Q1,Q3)	51 (45, 57)	56 (48.5, 60.5)
**CVLT-II**		
Mean (*SD*)	56.55 (11.29)	58.62 (8.60)
Median (Q1, Q3)	61 (46, 67)	62 (52.5, 63)
**BVMT-R**		
Mean (*SD*)	26.91 (7.30)	30.08 (4.35)
Median (Q1, Q3)	30 (22, 33)	31 (28.5, 32.5)
**T25FWT**		
Mean (*SD*)	4.66 (0.61)	4.80 (0.66)
Median (Q1, Q3)	4.36 (4.28, 5.08)	4.54 (4.35, 5.32)
**9HPT**		
Mean (*SD*)	20.33 (4.54)	18.66 (2.02)
Median (Q1, Q3)	19.28 (17.39, 20.57)	17.94 (17.11, 20.14)

*EDSS, Expanded Disability Status Score; MSSS, Multiple Sclerosis Severity Score; NAA-Cr, N-acetylaspartate/creatinine; CBF, Cerebral Blood Flow; FSS, Fatigue Severity Scale; MFIS, Modified Fatigue Impact Scale; FSMC, Fatigue Scale for Motor and Cognitive functions; BDI-II, Beck Depression Inventory-II; SDMT, Symbol Digit Modalities Test; CVLT-II, California Verbal Learning Test-II; BVMT-R, Brief Visuospatial Memory Test-Revised; T25FWT, Timed 25 Foot Walk Test; 9HPT, 9-Hole Peg Test*.

* *p < 0.05*.

**Figure 2:**
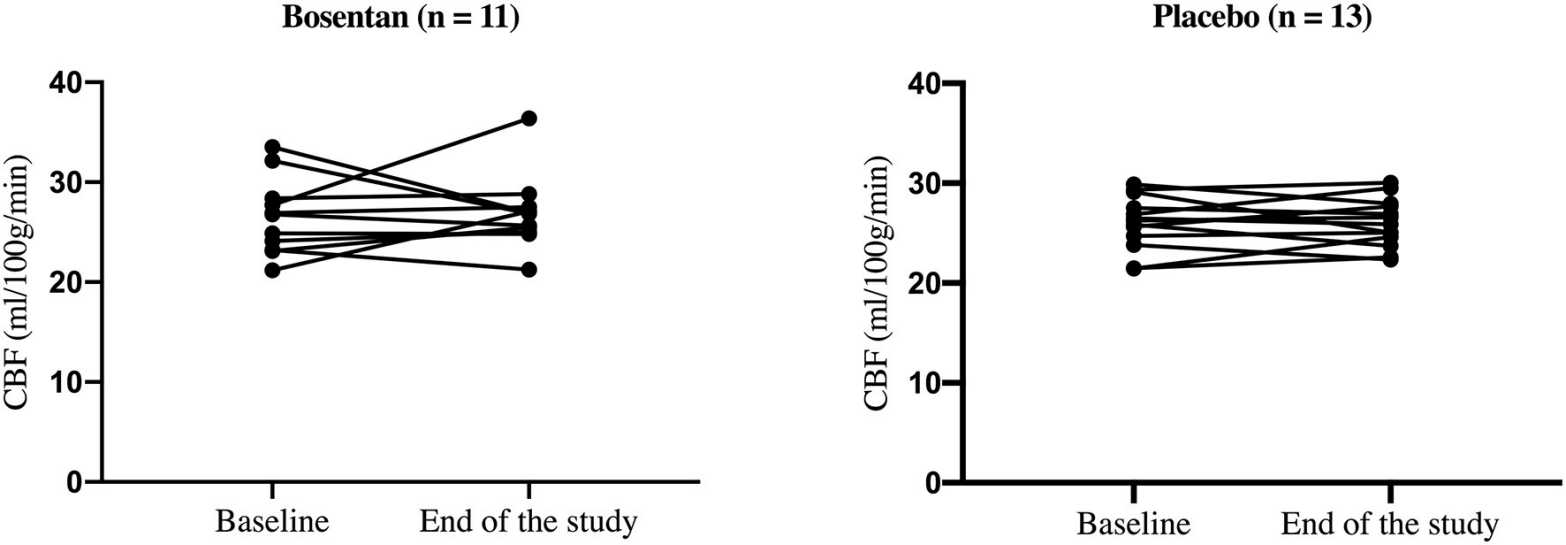
Individual CBF values in the centrum semiovale NAWM at baseline and end of study.

For the two treatment groups, there was no significant change in (mean ± SD) CBF values in centrum semiovale NAWM between baseline and end of the study in the bosentan group (26.5 ± 3.8 vs. 26.9 ± 3.7 ml/100 g/min) and the placebo group (26.0 ± 2.7 vs. 26.0 ± 2.4 ml/100 g/min). Individual values are shown in Figure 2.

No significant change was found in (mean ± SD) NAA/Cr ratio between baseline and end of the study in the bosentan group (2.00 ± 0.19 vs. 1.95 ± 0.18) and the placebo group (2.04 ± 0.19 vs. 2.09 ± 0.17).

For all secondary outcome parameters there were no differences between the two treatment groups over time.

The treatment was well-tolerated without side effects, with the exception of one patient in the placebo group who discontinued because of dizziness.

**Figure 3:**
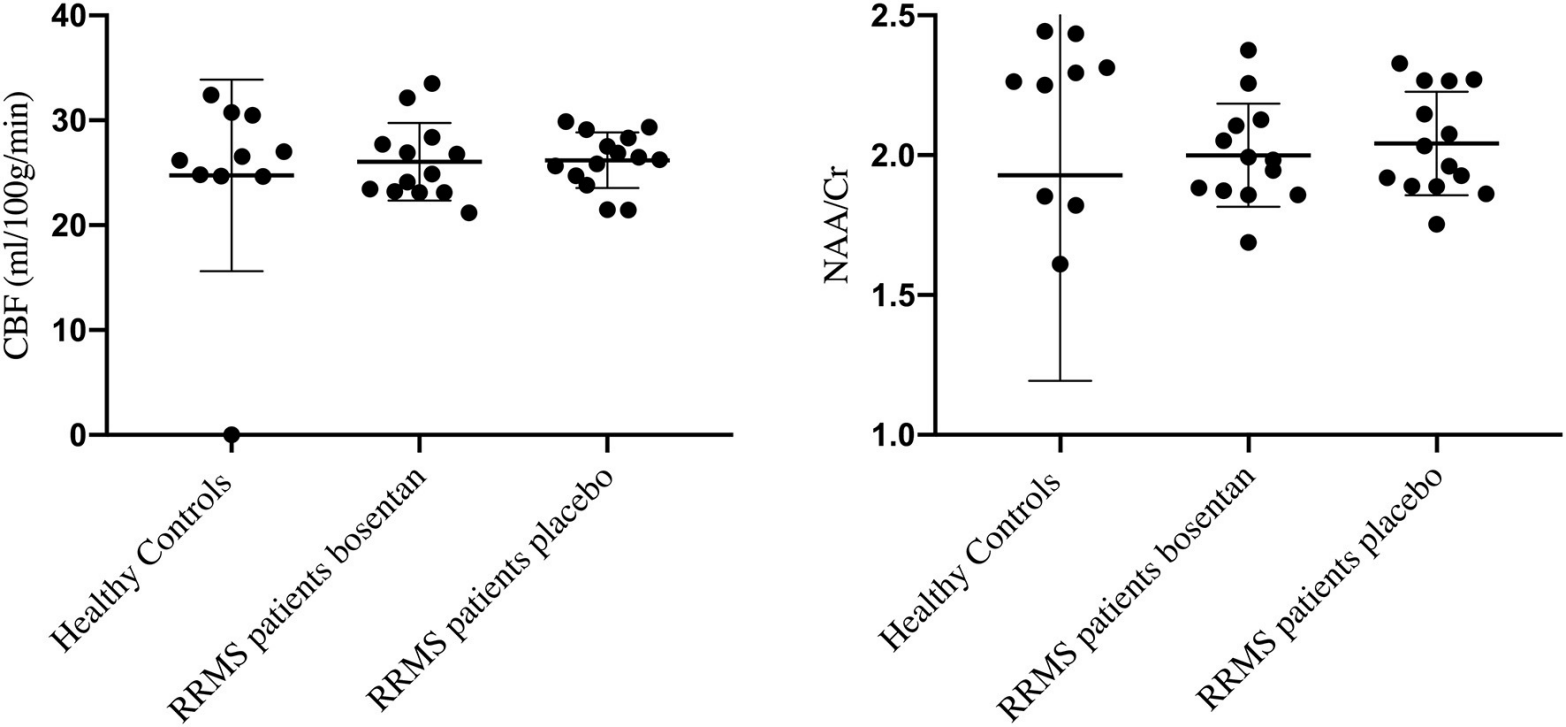
CBF values and NAA/Cr ratios in healthy controls and RRMS patients randomized to bosentan or placebo.

The mean (±SD) age of the healthy controls (38 ± 12 years) and gender balance (5 females/5 males) were not significantly different form the RRMS patients. There was no significant difference in (mean ± SD) CBF values in centrum semiovale NAWM at baseline between healthy controls (24.75 ± 9.13 ml/100 g/min) and the RRMS patients (26.12 ± 3.14 ml/100 g/min). No significant difference was found in (mean ± SD) NAA/Cr ratio at baseline between the healthy controls (1.93 ± 0.73) and the RRMS patients (2.02 ± 0.18) (Figure 3).

## Discussion

In this proof-of concept trial, we found no increase in CBF in NAWM of the centrum semiovale of RRMS patients treated for 28 days with the ET-1 antagonist bosentan. Accordingly, we did not find an effect on NAA levels in NAWM of the centrum semiovale and on the clinical parameters. This negative result was unexpected since D'Haeseleer et al. found that in MS patients, CBF increased to values similar to those in healthy control subjects after a single intake of one tablet of 62.5 mg bosentan (3). However, in that study, CBF in the centrum semiovale NAWM of the MS patients was about 20% lower than that measured in age and sex matched healthy controls. To the best of our knowledge, no other studies have investigated the effects of bosentan on CBF in MS patients.

In NAWM of the corpus callosum of MS patients, Saindane et al. found that the decreased CBF did not correlate with fractional anisotropy, suggesting that reduced perfusion in the NAWM of MS patients is not just a consequence of axonal degeneration (17). D'Haeseleer et al. found that ET-1 produced by reactive astrocytes in MS plaques is likely responsible for the reduced CBF in MS patients (3). They found that astrocytes in postmortem samples of cerebral white matter from individuals without neurological disease did not visually show ET-1 immunostaining, whereas reactive astrocytes in demyelinated plaques form patients with secondary progressive MS were strongly positive for ET-1. This ET-1 production leads to increased ET-1 levels in cerebrospinal fluid (CSF) and blood of patients with MS (3, 18–20). However, significance is at group level, and these studies show that there are also MS patients who, at the moment of sampling, have no increased ET-1 levels in CSF or blood. ET-1 levels were significantly increased in CSF of RRMS patients with an acute clinical attack in comparison with those in a stable phase (18). Rocha et al. found no association between plasma ET-1 levels and peripheral immune markers (20). However, we recently showed that the pro-inflammatory cytokines IL-1β and TNF-α, which are present in active inflammatory MS plaques, stimulated ET-1 secretion in cultured human astrocytoma cells (21). All together, these findings indicate that local inflammatory mechanisms in MS plaques are likely the trigger for the astrocytic ET-1 production that is responsible for lowering CBF in patients with MS.

We found that CBF in the centrum semiovale NAWM in our RRMS population was not different from that of healthy controls who were investigated with the same cerebral MRI protocol, software and scanner. After the start of our clinical trial, a number of studies reported that in MS patients there is a relationship between disease severity and the degree of cerebral hypoperfusion (22, 23). The patients in our trial were only mildly disabled (low EDSS scores and normal NAA/Cr ratios), and most were satisfactorily treated with immunomodulatory drugs, suggesting that their normal CBF values were related to minimal focal inflammatory activity in their CNS, so that there is no significant astrocytic production of ET-1. A major limitation of our study is that we did not measure ET-1 levels in blood or CSF.

Subarachnoid perivascular micro-application of bosentan on individual pial arterioles on the cortical surface of anesthetized cats only had minimal effect on pial arteriolar caliber. Subarachnoid perivascular micro-application of ET-1 effected a marked reduction in pial arteriolar caliber, which could be attenuated either by co-administration of bosentan or by the intravenous administration of bosentan (24). The administration of intravenous bosentan in anesthetized pigs also did not significantly change CBF measured with positron emission tomography (25). In a rat model of pneumococcal meningitis, ET-1 levels increased five-fold in the CSF, and CBF was significantly reduced by about 50%. CBF was restored to levels similar to controls after intraperitoneal administration of bosentan (26). All these data indicate that bosentan only has a significant effect on the CBF in conditions associated with local ET-1 production causing some degree of vasoconstriction of the cerebral vasculature.

Astrocytic ET-1 production and decreased CBF may be an important modifiable aggravating factor for disease progression in MS patients, but not all, as our study has shown. A recent study showed that significantly higher CSF levels of ET-1 were found in MS patients with aggressive optic neuritis (ON) compared to non-aggressive ON (27). MS patients with decreased CBF may benefit from therapies blocking astrocytic ET-1 production or ET-1 antagonists. However, future clinical trials assessing possible beneficial effects of bosentan in patients with MS should only include patients with increased blood or CSF levels of ET-1.

## Data Availability Statement

The datasets generated for this study are available on request to the corresponding author.

## Ethics Statement

The studies involving human participants were reviewed and approved by this study was registered by the European Union Drug Regulating Authorities (Eudra-CT 2017-001253-13) and was approved at national level by the Federaal Agentschap Voor Geneesmiddelen en Gezondheidsproducten (FAGG, Belgium) as well as by the ethics committee of the UZ Brussel. The patients/participants provided their written informed consent to participate in this study. Written informed consent was obtained from the individual(s) for the publication of any potentially identifiable images or data included in this article.

## Author Contributions

SH, MD'H, and JD designed the ROCHIMS trial. WC provided statistical advice. A-MV, PV, and HR performed the MRI studies. SH performed the clinical trial, analyzed the data, and wrote the manuscript. JD and MD'H made critical revisions to the manuscript. All authors discussed the results and read and approved the final version of the manuscript. All authors contributed to the article and approved the submitted version.

## Conflict of Interest

The authors declare that this study received funding/study components from Actelion Pharmaceuticals Belgium NV. The funder was not involved in the study design, collection, analysis, interpretation of data, the writing of this article, or the decision to submit it for publication.
